# A Review for Compact Model of Thin-Film Transistors (TFTs)

**DOI:** 10.3390/mi9110599

**Published:** 2018-11-15

**Authors:** Nianduan Lu, Wenfeng Jiang, Quantan Wu, Di Geng, Ling Li, Ming Liu

**Affiliations:** 1 Key Laboratory of Microelectronic Devices & Integrated Technology, Institute of Microelectronics, Chinese Academy of Sciences, Beijing 100029, China; lunianduan@ime.ac.cn (N.L.); jiangwenfeng1912@163.com (W.J.); wuquantan@ime.ac.cn (Q.W.); gengdi@ime.ac.cn (D.G.); liuming@ime.ac.cn (M.L.); 2 School of Microelectronics, University of Chinese Academy of Sciences, Beijing 100049, China; 3 Jiangsu National Synergetic Innovation Center for Advanced Materials (SICAM), Nanjing 210009, China

**Keywords:** thin-film transistors (TFTs), compact model, surface potential

## Abstract

Thin-film transistors (TFTs) have grown into a huge industry due to their broad applications in display, radio-frequency identification tags (RFID), logical calculation, etc. In order to bridge the gap between the fabrication process and the circuit design, compact model plays an indispensable role in the development and application of TFTs. The purpose of this review is to provide a theoretical description of compact models of TFTs with different active layers, such as polysilicon, amorphous silicon, organic and In-Ga-Zn-O (IGZO) semiconductors. Special attention is paid to the surface-potential-based compact models of silicon-based TFTs. With the understanding of both the charge transport characteristics and the requirement of TFTs in organic and IGZO TFTs, we have proposed the surface-potential-based compact models and the parameter extraction techniques. The proposed models can provide accurate circuit-level performance prediction and RFID circuit design, and pass the Gummel symmetry test (GST). Finally; the outlook on the compact models of TFTs is briefly discussed.

## 1. Introduction

**Figure 1:**
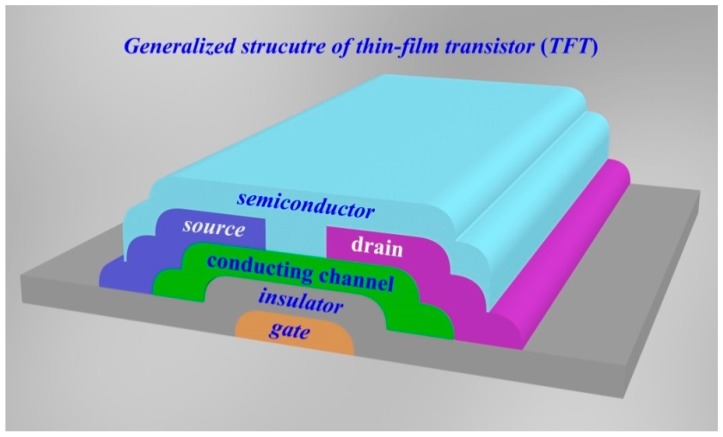
Schematic structure of a generalized thin-film transistor.

A thin-film transistors (TFTs) is a special kind of field-effect transistor (FET) fabricated by depositing thin films of an active semiconductor layer, as well as the dielectric layer and metallic contacts over a supporting (but non-conducting) substrate [1,2]. In the past 15 years, TFTs has grown into a huge industry based on display, memory, E-paper applications, and so on [3,4,5,6]. Generally, a common substrate in TFTs is glass, which differs from the conventional transistor, where the semiconductor material typically is the substrate, such as a silicon wafer. TFTs include three basic elements: (1) a thin semiconductor film; (2) an insulating layer; and (3) three electrodes (gate, source and drain) [7,8,9]. Three basic elements for configuration of TFT have been illustrated clearly in Figure 1. The source and drain, are in contact with the semiconductor film at a short distance from one another. The gate is separated from the semiconductor film by the insulating layer [10].

The history of TFT really began with the work of P. K. Weimer at Radio Corporation of America (RCA) Laboratories in 1962 [11]. At that time Weimer fabricated the first TFT based on thin films of polycrystalline cadmium sulfide as the semiconductor materials. In the 1970s, the realization of crystalline silicon as the active materials with low cost dramatically changed the prospects of TFTs [12]. In 1979, amorphous silicon as a new active material was introduced by LeComber et al. [13], which had profound implications for TFTs. In 1980, Depp et al. reported polysilicon TFT which achieved good mobility and TFT characteristics [14]. In 1986, the first transistor based on organic semiconductor was reported [15]. As compared with conventional Si TFTs, organic TFT (OTFT) displays much less complex in fabrication processes and can be naturally compatible with plastic substrates for lightweight and foldable products [16]. To develop large-scale TFTs, processing temperatures must be getting lower and lower. In 2004, Nomura et al. used a complex In-Ga-Zn-O (IGZO) semiconductor layer in a TFT, which achieved the room-temperature processing of the semiconductor layer [17]. Looking back into the past half-century, TFTs moved endlessly forward from the initial requirement of performance to today’s application of large area and low cost.

During the development of TFTs, the semiconductor device model represents an essential bridge between the semiconductor manufactures and the circuit design. Integrated circuit (IC) designers usually utilize various kinds of software (such as Cadence, SPICE, PHILIPAC) for design circuit [18,19,20]. The core of the corresponding software is the model of each unit device. Because the IC is consisted of several transistors, if all unit devices would need to run the complicated model of transistor, the system level simulation will beyond computer ability and hence causes non-convergence in calculation. Otherwise, for ensuring the reliability of the simulation, the device model should also be able to accurately describe the physical properties [21]. Compact model is a critical step in the design cycle of modern IC products [22]. It refers to the development of models for integrated semiconductor devices for use in circuit simulations. Compact model is usually used to reproduce device terminal behaviors with accuracy, computational efficiency, ease of parameter extraction, and relative model simplicity for a circuit or system-level simulation, for future technology nodes [23].

Accurate and physical compact models are essential for digital and analog circuits. Generally speaking, an excellent compact model should include the following requirements [21,24]:(i) Representing consistently the behavior; (ii) Being symmetrical to reflect the symmetry of TFT structure; (iii) Being analytical, without differentials or integrals; (iv) Being simple and easily derivable; (v) Parameters that can be characterized easily, or even guessed; (vi) Being upgradable and reducible; (vii) Relations can be physically justified; (viii) Being similar form and correspondence to compact models for other TFTs; (ix) Being tunable to inaccurate (or uncertain) experimental data.

The first compact model could date back to 1983, in which Kacprzak et al. proposed a compact DC model of GaAs FETs for large-signal computer calculation [25]. In 1986, based on one-dimensional (1-D) solution of Poisson’s equation, Ahmed et al. reported a compact model for accumulation mode poly-Si devices [26]. Later, plenty of methods, such charge sheet model, effective medium approach (EMA), semi-empirical approach, generation-recombination model, and surface-potential based model, have been introduced for the compact models of the silicon-based TFTs [27,28,29,30,31,32]. Then, with the emergence of new TFTs, e.g., OTFT and IGZO TFTs, some excellent compact models based on interesting methods have been developed [33,34,35,36,37]. Strictly speaking, all of the proposed compact models can be divided into two categories. One is charge-based and another is surface-potential-based. As compared with the charge-based model, the surface-potential-based compact model is believed to have high accuracy and strong physical property, and be easily simplified into the charge-based and threshold-voltage-based model [21]. It can also describe the operation of transistor more accurately without any smooth functions [38].

Over the past two decades, although some excellent reviews have been published [39,40,41,42], a completed review for the compact models of TFTs based on different active materials is still lacking. In this review, we will provide an updated review of surface-potential-based compact model of TFTs with different active materials, such as polysilicon, amorphous silicon, organic and IGZO semiconductors. In Section 2, the charge transport property of different active materials is discussed. In Section 3, we discuss the surface-potential-based compact models for silicon-based TFTs and presented our surface-potential-based compact models for organic and IGZO TFTs, respectively. In Section 4, the comparison of various compact models will be summarized. Finally, the future outlook for this field is briefly discussed in Section 5.

## 2. Charge Transport Property

In order to achieve an accurate compact model for TFTs, the key is to correctly describe the charge transport characteristics. For TFTs with different active materials, the charge transport has displayed various properties. This section will, in detail, introduce the charge transport properties of TFTs for different active materials.

### 2.1. Grain-Boundary Trapping Theory

Based on its structure characteristics, the charge transport property of polysilicon has been described in terms of two distinct models: segregation theory and grain-boundary trapping theory [43]. In the segregation theory, impurity atoms tend to segregate at the grain boundary where they are electrically inactive. While the grain boundary trapping theory assumed that the presence of a large amount of trapping states at the grain boundary able to capture, and therefore immobilize, free carriers. The basic limitation of segregation theory is that it does not explain the temperature dependence of the film resistivity which is thermally activated and exhibits a negative temperature coefficient. The grain-boundary trapping theory can explain most of electrical properties in polysilicon.

**Figure 2:**
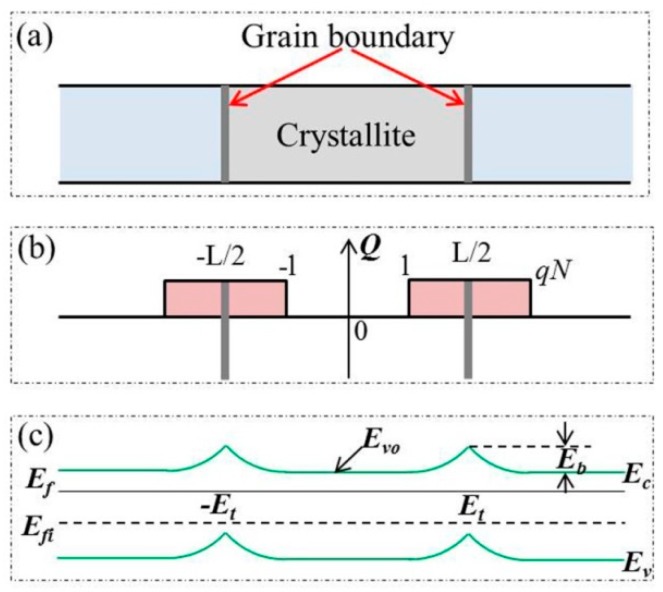
Schematic diagram of (**a**) crystal structure; (**b**) charge distribution; and (**c**) energy band structure of polysilicon films.

In the grain-boundary trapping theory, a polysilicon is assumed to be composed of small crystallites joined together by the grain boundaries usually consisted of a few atomic layers of disordered atoms [43]. Inside each crystallite the atoms are arranged in a periodic manner so that it can be considered as a small single crystal. Atoms in the grain boundary represent a transitional region between the different orientations of neighboring crystallites. Although polysilicon is a three-dimensional substance, it is sufficient to treat the problem in one dimension to calculate the transport properties. The traps are assumed to be initially neutral and become charged by trapping a carrier. Figure 2 shows the schematic diagram of crystal structure, charge distribution and energy band structure of polysilicon films.

The grain-boundary trapping theory considers just the resistance of the grain-boundary region, which includes two important contributions to the current: thermionic emission and tunneling (field emission) [44]. Thermionic emission results from those carriers possessing high enough energy to surmount the potential barrier at the grain boundary. The tunneling current arises from carriers with energy less than the barrier height. When the barrier is narrow and high, the tunneling current can become comparable to or larger than the thermionic emission current. In the polysilicon the potential barrier is the highest when the barrier width is the widest. Because of this, tunneling current may be neglected. Then, for an applied voltage the thermionic emission current density across a grain boundary following Bethe is expressed as the following [44]:

(1) $$J_{th}=qp_{a}{\left(\frac{k_{B}T}{2m^{*}\pi }\right)}^{1/2}exp\left(-\frac{qV_{B}}{k_{B}T}\right)\left[exp\left(-\frac{qV_{a}}{k_{B}T}\right)-1\right]$$

where *q* is the elemental charge, $p_{a}$ is the average carrier concentration, $m^{*}$ is the effective mass of the carrier, $k_{B}$ is the Boltzmann constant, $V_{B}$ is the potential barrier height, and $V_{a}$ is the applied voltage. Equation (1) neglects collisions within the depletion region and the carrier concentration in the crystallite was assumed to be independent of the current flow, so that it is applicable only if the number of carriers taking part in the current transport is small compared to the total number of carriers in the crystallite. This condition restricts the barrier height to be larger than or comparable to $k_{B}T$. If $V_{a}$ is small, $qV_{a}\ll k_{B}T$, Equation (1) can be expanded to give the following:

(2) $$J_{th}=q^{2}p_{a}{\left(\frac{1}{2\pi m^{*}k_{B}T}\right)}^{1/2}exp\left(-\frac{qV_{B}}{k_{B}T}\right)V_{a}$$

which is a linear current–voltage relationship. Based on Equation (2), the conductivity of a polysilicon film with a grain size *L* is written as:

(3) $$\sigma =Lq^{2}p_{a}{\left(\frac{1}{2\pi m^{*}k_{B}T}\right)}^{1/2}exp\left(-\frac{qV_{B}}{k_{B}T}\right)$$

Then, the effective mobility is expressed as:

(4) $$\mu_{eff}=Lq{\left(\frac{1}{2\pi m^{*}k_{B}T}\right)}^{1/2}exp\left(-\frac{E_{b}}{k_{B}T}\right)$$

here $E_{b}$ is the energy barrier.

### 2.2. Hopping Transport

**Figure 3:**
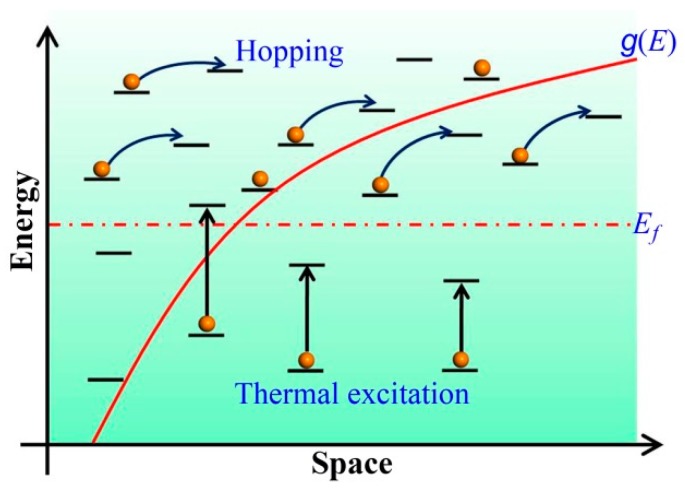
Schematic diagram of hopping transport with the density of states.

Differing from crystalline materials, such as polysilicon, the charge transport in amorphous materials exhibits very different properties. Amorphous semiconductor materials, including inorganic and organic, have in common, that their atomic or molecular structure is completely disordered. For inorganic amorphous semiconductors, such as, pure and hydrogenated amorphous silicon (a-Si, a-Si:H), a band structure similar to the one of crystalline materials still exists [45,46]. The electronic states in the conduction and valence bands are therefore delocalized. Thus some of the concepts from crystalline semiconductor physics are still suitable for the inorganic amorphous materials. However, in the band gap between valence and conduction band, some localized states exist in which charge carriers can be trapped. For organic amorphous semiconductors, the intermolecular bonds are due to relatively weak van der Waals interactions, the electronic wave functions usually do not extend over the entire volume of the organic solid, but rather, are localized to a finite number of molecules, or even to individual molecules [47,48]. Due to the spatial and energetic disorder, the charge transport in amorphous semiconductor materials is limited by trapping in the localized states. This means that the charge carrier mobility is expected to be thermally activated, that is, the charge transport always happens to jump from one localized site to another. This type of transport mechanism is called hopping transport. The transition of hopping between two sites depends on the overlap of the electronic wave functions of these two sites [49]. Whenever a charge carrier hops to a site with a higher (lower) site energy than the site that it came from, the difference in energy is accommodated for by the absorption (emission) of a phonon. Figure 3 is a schematic diagram of carrier hopping transport with the density of states [50].

The intrinsic transition rate for a carrier hopping from an initial site *i* to an empty site *j* is expressed by $\gamma_{ij}=\gamma \left(R_{ij},\;E_{i}-E_{j}\right)$. The average transition rate from site *i* to site *j* is then [51]:

(5) $$\nu_{ij}=<m_{i}\left(1-m_{j}\right)\;\gamma_{ij}>$$

where $m_{i}$ and $m_{j}$ are the occupation numbers for sites *i* and *j*, respectively. The energy dependence of $\gamma_{ij}$ is then a good approximation to take the Miller–Abrahams form to write as [52]:

(6) $$\gamma_{ij}=v_{0}exp\left(-2\varphi R_{ij}-\frac{\theta \left(E_{j}-E_{i}\right)}{k_{B}T}\right)$$

where $v_{0}$ is the attempt-to-jump frequency, φ is the inverse localized length of the inverse wave function, $R_{ij}$ is the distance between site *i* and site *j*, $E_{i}$ and $E_{j}$ are the energies of sites *i* and *j*, respectively, and θ(x)=xε(x) with ε(x) being the step function.

### 2.3. Multiple Trapping and Release Theory

**Figure 4:**
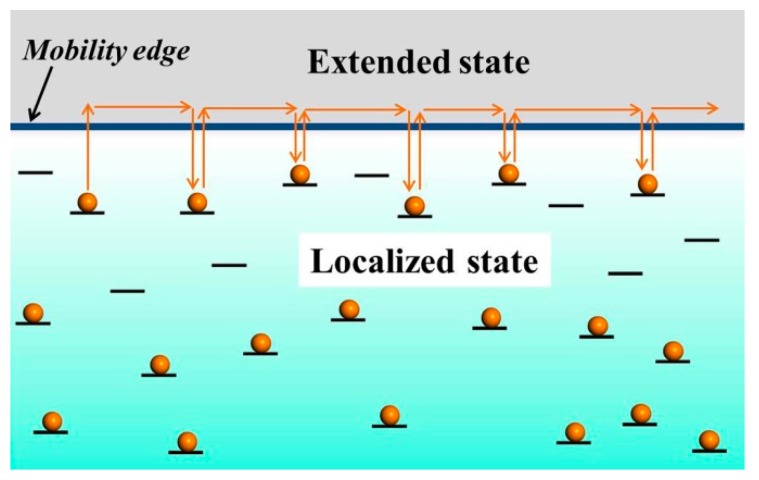
Transport diagram of multiple trapping and release (MTR) theory. The charge carrier (orange balls) is trapped and released into and from localized states (black lines). Conduction happens above the mobility-edge (gray area).

For some special materials, such as the small-molecule organic semiconductor and the IGZO semiconductor, which have a strong tendency to form polycrystalline films [50,53,54]. These semiconductors display the regular arrangement, and the delocalized orbitals partially overlap, thereby facilitating more efficient charge transfer and carrier mobility that is much larger than in amorphous films. The charge transport properties of these materials cannot be explained by the grain-boundary trapping theory and hopping transport. In contrast to the grain-boundary trapping theory or hopping theory, the multiple trapping and release (MTR) theory is adapted for the materials [55,56]. MTR theory assumes that the charge transport occurs in extended states, and that most of the charge carriers are trapped in localized states [57]. Energy of localized state is separated from mobility edge energy. When the energy of localized state is slightly lower mobility edge, then the extended states acts as shallow trap, from which the charge carrier can be released (emitted) by the thermal excitations. But, if that energy is far below mobility edge energy, then charge carriers cannot be thermally excited (emitted). The number of carriers available for transport depends on the difference in energy between the trap level and the extended-state band. Figure 4 is a transport diagram of MTR theory.

In the MTR theory, total charge carriers’ densities, $n_{total}$, is equal to sum of density in extended states, $n_{e}$, and in localized states, as in Ref. [57]:

(7) $$n_{total}=n_{e}+\int_{-\infty }^{0}g\left(E\right)f\left(E\right)dE$$

where the upper limit of integral *E* = 0 corresponds to the mobility edge, g(E) is the trap density of states (DOS) energy distribution. $f\left(E\right)={\left(1+exp\left(\frac{E-E_{f}\left(x\right)}{k_{B}T}\right)\right)}^{-1}$ is the Fermi–Dirac distribution, $E_{f}\left(x\right)$ is the quasi-Fermi level. Two methods within the MTR theory usually describe the effect of trapping [58]. One is that, all carrier fields induced can contribute to the current flow at any moment of time, but the effective mobility is reduced in comparison with its intrinsic, trap-free value:

(8) $$\mu_{eff}=\mu_{0}\left(T\right)\frac{\tau \left(T\right)}{\tau \left(T\right)+\tau_{tr}\left(T\right)}$$

here, $\mu_{0}$ is the carrier mobility in extended state, $\tau_{tr}\left(T\right)$ is the average trapping time on shallow traps, and τ(T) is the average time that a polaron spends diffusively traveling between the consecutive trapping events. Another is that only a fraction of the carrier field induced is moving at any given moment of time:

(9) $$n_{eff}=n_{total}\frac{\tau \left(T\right)}{\tau \left(T\right)+\tau_{tr}\left(T\right)}$$

## 3. Surface-Potential-Based Compact Models

In spite of the fact that the transport characteristics in TFTs is very different for different active materials, the current–voltage characteristics can, to first order, be described with the same formalism as [53]:

(10) $$I_{ds}=\{\begin{array}{l} \frac{\mu C_{i}W}{L}\left(\left(V_{g}-V_{th}\right)V_{ds}-\frac{V_{ds}^{2}}{2}\right)\;\;\;\;\;\;for\;\left|V_{g}-V_{th}\right|>\left|V_{ds}\right|\;\left(linear\;regime\right)\\ \frac{\mu C_{i}W}{2L}{\left(V_{g}-V_{th}\right)}^{2}\;\;\;\;\;\;\;\;for\;\left|V_{ds}\right|>\left|V_{g}-V_{th}\right|>0\;\left(saturation\;regime\right) \end{array}$$

where Equation (10) describes the relationship between the drain current $I_{ds}$, the gate-source voltage $V_{g}$ and the drain-source voltage $V_{ds}$ in linear and saturation regimes, respectively. $C_{i}$ is the gate dielectric capacitance per unit area, μ is the carrier mobility in the semiconductor, W and L is the channel width and length of the transistor, respectively. For silicon TFTs, the threshold voltage $V_{th}$ is defined as the minimum gate-source voltage required to induce strong inversion [59]. However OTFTs and IGZO TFTs usually operate in accumulation region, thus strictly speaking the threshold voltage cannot be defined for OTFTs and IGZO TFTs. Since the threshold voltage concept is nonetheless useful, the compact models will show very different for TFTs with different active materials. Otherwise, the central aim of compact models is to accurately and physically describe the current–voltage characteristics of TFTs in Equation (10). As mentioned above, the surface-potential-based compact model is believed to have high accuracy and strong physical properties. The following will review the surface-potential-based compact models for polysilicon and amorphous silicon TFTs, and then present our compact models for OTFTs and IGZO TFTs based on surface-potential-based.

### 3.1. Polysilicon TFT Compact Models

Polysilicon TFTs have gotten considerable applications, especially in active matrix liquid crystal displays (AMLCDs), printers, scanners, Static Random-Access Memories (SRAMs) and three-dimensional large scale integration (LSI) circuits [60]. In early time, researchers usually built the polysilicon TFT models based on the one-dimensional solution of Poisson’s equation and the effects of grain-boundary traps [26,61]. However, these earlier models were unclear for inversion mode devices due to the “reverse” charge shielding concept defined in its derivation [62]. Later, some authors adopted the EMA method to well address the question of non-uniform polysilicon sample with the grain boundaries [29,63]. In 1999, Benjamín et al. also adopted EMA to develop a unified model for long and short-channel polysilicon TFTs [28]. This method is attractive because it accounts for field effect mobility enhancement in the moderate inversion regime and for mobility degradation at high gate voltages, for drain-induced barrier lowering (DIBL) effect, kink effect, off-state current and channel-length modulation. A few years later, Wu et al. proposed a compact model by approximating the generation rate for poly Si TFTs in the leakage region [50]. Although several models for poly-Si TFTs have been proposed so far, based on different equations for the subthreshold, linear, and saturation regions [64,65], these methods always lead to a significant error in evaluating derivatives such as transconductance [66].

To capture more accurate features of poly-Si TFTs, Shimizu et al. developed a compact model based on a new surface-potential-based [67]. Firstly, in the model the states are approximated by the sum of exponential distributions for the deep and tail states as:

(11) $$g\left(E\right)=g_{de}exp\left(\frac{E-E_{c}}{E_{de}}\right)+g_{ta}exp\left(\frac{E-E_{c}}{E_{ta}}\right)$$

where $E_{de}$ and $E_{ta}$ are the inverse slope of deep states and tail states, respectively, $g_{de}$ and $g_{ta}$ are the density of deep state and tail state at bottom of conduction band $E_{c}$, respectively.

By integrating the 1-D Poisson equation, the surface potentials at the source side as a function of gate voltage can be calculated numerically as the following [68]:

(12) $$C_{i}\left(V_{g}-V_{fb}-\varphi_{s0}\right)=\sqrt{\frac{2q\varepsilon_{s}N_{sub}}{\beta }}{\left[exp\left(-\beta \varphi_{s0}\right)-exp\left(-\beta \varphi_{b0}\right)+\beta \left(\varphi_{s0}-\varphi_{b0}\right)+{\left(\frac{n_{i}}{N_{sub}}\right)}^{2}\left[exp\left(\beta \varphi_{s0}\right)-exp\left(\beta \varphi_{b0}\right)\right]+\left(\frac{\beta N_{deep}}{\gamma N_{sub}}\right)\left[exp\left(\gamma \varphi_{s0}\right)-exp\left(\gamma \varphi_{b0}\right)\right]+\left(\frac{N_{tail}}{N_{sub}}\right)\left[exp\left(\beta \varphi_{s0}\right)-exp\left(\beta \varphi_{b0}\right)\right]\right]}^{\frac{1}{2}}$$

where $\gamma =q/E_{de}$, β is the inverse of thermal voltage, $\varepsilon_{s}$ is the dielectric constant, $N_{sub}$ is the dopant concentration, $n_{i}$ is the intrinsic carrier concentration, $\varphi_{s0}$ and $\varphi_{b0}$ are the front and back surface potentials at the source side, respectively, $N_{deep}$ and $N_{tail}$ are the densities of trapped electrons in deep states and tail states under a flat band condition, respectively.

Then, the inversion layer charge density at the source (*x* = 0) or drain (*x* = *L*) side can be written as [69] the following:

(13) $$Q_{i}\left(x\right)=-C_{i}\left(V_{g}-V_{fb}-\varphi_{s0}\right)+\sqrt{\frac{2q\varepsilon_{s}N_{sub}}{\beta }}{\left[exp\left(-\beta \varphi_{sx}\right)-exp\left(-\beta \varphi_{bx}\right)+\beta \left(\varphi_{sx}-\varphi_{bx}\right)+\;\left(\frac{\beta N_{deep}}{\gamma N_{sub}}\right)\left[exp\left(\gamma \varphi_{sx}\right)-exp\left(\gamma \varphi_{bx}\right)\right]+\left(\frac{N_{tail}}{N_{sub}}\right)\left[exp\left(\beta \varphi_{sx}\right)-exp\left(\beta \varphi_{bx}\right)\right]\right]}^{\frac{1}{2}}$$

**Figure 5:**
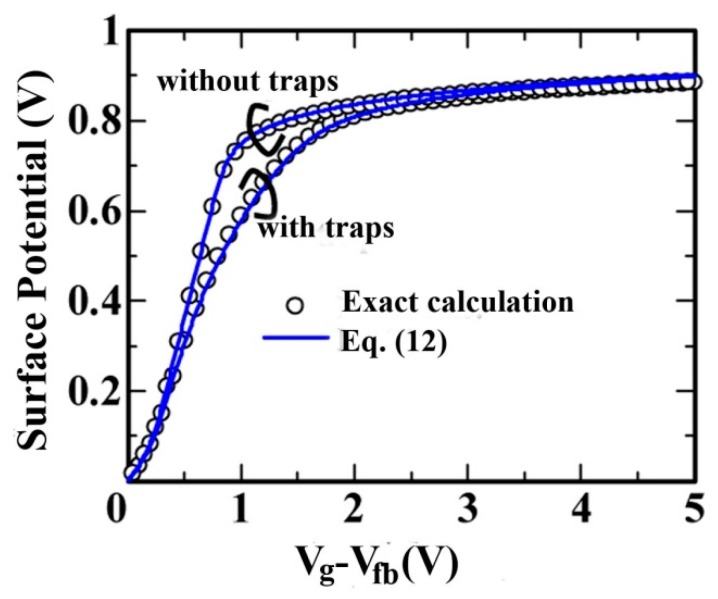
Comparison of calculated front surface potential obtained using Equation (12) (lines) and the exact numerical calculations (circles) with and without traps as a function of gate voltage.

Obviously, Equations (12) and (13) could be only solved by iteration. To determine the surface potentials at the source side or at the drain side, the authors used a method from the literature [70]. In Equation (13), the charge densities of inversion layer are derived based on the charge-sheet approximation. Figure 5 shows a comparison of the front surface potentials obtained from Equation (12) and the exact numerical calculations.

After obtaining the surface potentials, based on the drift-diffusion approximation [71], the authors calculated the drain current as:

(14) $$I_{ds}=\frac{W\mu }{L\beta }\left[C_{i}\left(\beta \left(V_{g}-V_{fb}\right)+1\right)\left(\varphi_{sL}-\varphi_{s0}\right)-\frac{\beta }{2}C_{i}\left(\varphi_{sL}^{2}-\varphi_{s0}^{2}\right)-\frac{\beta }{2}\left(q_{i}\left(0\right)+\left(L\right)\right)\left(\varphi_{sL}-\varphi_{s0}\right)-\left(q_{i}\left(0\right)-q_{i}\left(L\right)\right)\right]$$

where $\varphi_{sL}$ and $\varphi_{bL}$ are the front and back surface potentials at the drain side, respectively, $q_{i}\left(x\right)=Q_{i}\left(x\right)+C_{i}\left(V_{g}-V_{fb}-\varphi_{sx}\right)$. Note, Equation (14) can describe the drain current in all the regions of operation using the unified equation. At the same time, the model did not include the threshold voltage.

**Figure 6:**
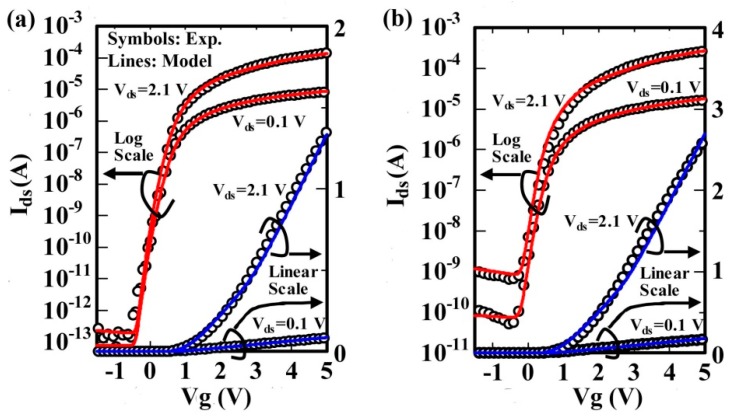
Comparison of measured (circles) and simulated (lines) drain current characteristics as a function of gate voltage on logarithmic (left axis) and linear (right axis) scales for an n-channel poly-Si thin-film transistors (TFT) with (**a**) W/L = 2 μm/2 μm and (**b**) W/L = 2 μm/1 μm.

**Figure 7:**
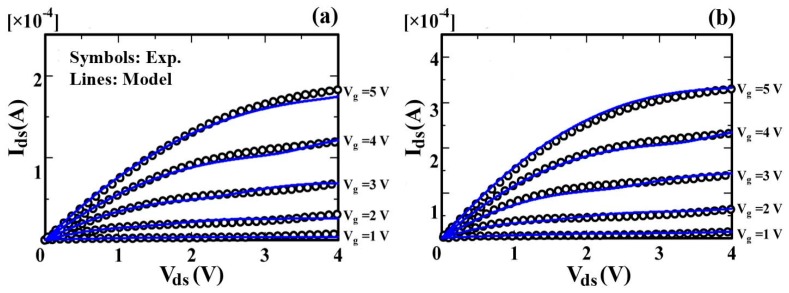
Comparison of measured (circles) and simulated (lines) drain current characteristics as a function of drain voltage for an n channel poly-Si TFT with (**a**) W/L = 2 μm/2 μm and (**b**) W/L = 2 μm/1 μm. The parameters used in the simulation are the same as those used in Figure 6.

Figure 6 shows a comparison of simulated and measured drain current characteristics as a function of gate voltage for an n-channel poly-Si TFT in the subthreshold and above-threshold regions. In the linear and saturation regions, a comparison of simulated and measured drain current characteristics is shown in Figure 7.

Differing from iterative solution of the surface potential in Shimizu et al.’s model, Chen et al. have developed an analytical solution to the surface potential of poly-Si TFTs by using the Lambert W function [72]. In Chen et al.’s model, the surface potential of poly-Si TFTs can be expressed as,

(15) $${\left(V_{g}-V_{fb}-\varphi_{s}\right)}^{2}=\gamma^{2}\left[\left(1+\frac{N_{T}}{L_{g}N_{A}}\right)\varphi_{s}+\varphi_{t}exp\left(\frac{\varphi_{s}-\varphi_{n}-2\varphi_{f}}{\varphi_{t}}\right)\right]-\frac{N_{T}}{L_{g}N_{A}}\varphi_{t}\ln\left(1+K_{m}\right)\;$$

where $\varphi_{s}$ is the surface potential, γ denotes a body factor: $\gamma =\sqrt{2\varepsilon_{s}qN_{A}}/C_{i}$, $L_{g}$ is the grain size, $N_{T}$ and $N_{A}$ are located traps and acceptor density, respectively, $\varphi_{t}$ is the thermal voltage, $\varphi_{f}$ is the Fermi potential, $\varphi_{n}$ is the channel voltage, $K_{m}=0.5exp\left(\frac{E_{t}+q\varphi_{t}}{k_{B}T}\right)$. To derive an analytical and non-iterative evaluation, the normalized form of Equation (15) can be written as the following:

(16) $${\left(v_{g}-x_{W}\right)}^{2}=G_{TFT}^{2}\left[x_{W}+\Delta_{TFT}\exp\left(x_{W}\right)+A\right]$$

where $x_{W}=\varphi_{s}/\varphi_{t}$ is the normalized surface potential, $v_{g}=\left(V_{g}-V_{fb}\right)/\varphi_{t}$ the normalized effective gate voltage, $G_{TFT}=\gamma \sqrt{\frac{1+N_{T}/L_{g}N_{A}}{\varphi_{t}}}$, $\Delta_{TFT}=\exp\left(\frac{-\varphi_{t}-2\varphi_{f}}{\varphi_{t}}\right)/(1+\frac{N_{T}}{L_{g}N_{A}})$, and $A=-\frac{N_{T}\ln\left(1+K_{m}\right)}{N_{T}+L_{g}N_{A}}$.

Then, with a simple mathematical procedure and using the principal branch of the Lambert W function [73], the authors obtained the physics-based analytical solution of the normalized surface potential as follows:

(17) $$x_{W}=-W_{0}\left[f\times \Delta_{TFT}exp\left(v_{G}-f\times A\right)\right]+v_{G}-f\times A$$

where $v_{G}=\left(v_{g}+\frac{G_{TFT}^{2}}{2}\right)-G_{FET}\sqrt{v_{g}+G_{TFT}^{2}/4}$ and $f=G_{FET}/2\sqrt{v_{g}+G_{TFT}^{2}/4}$.

In order to improve the accuracy, some corrections by using the Schroder series in the surface potential expression have been provided for Equation (17). Finally, the complete solution to the physics-based surface potential of poly-Si TFTs with absolute error only in nanovolt range can be expressed as:

(18) $$\varphi_{s}=\left[x_{W}+\omega \left(y_{W},y_{W}^{\prime },y_{W}^{\prime \prime }\right)+\varepsilon \right]\varphi_{t}\;$$

**Figure 8:**
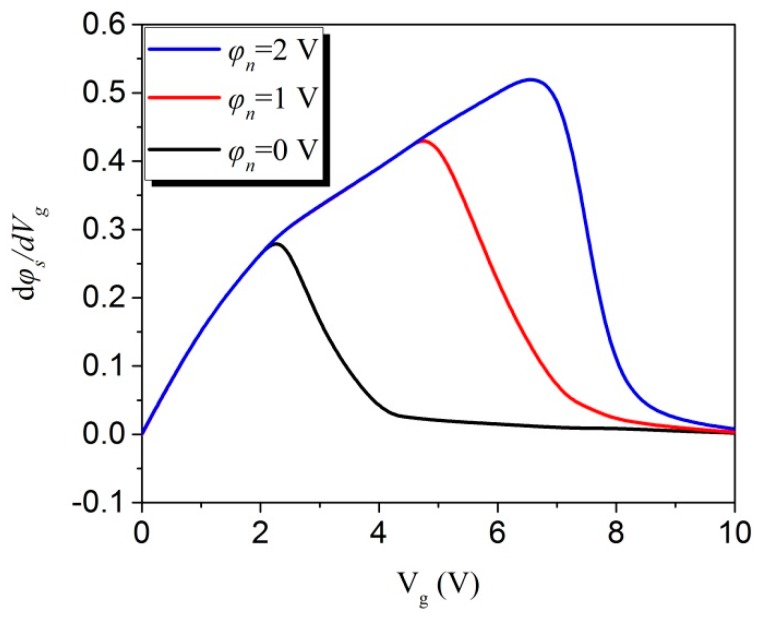
The characteristics of the surface potential derivative with respect to the gate voltage for different channel potential $\varphi_{n}$.

Based on Equation (18), the surface potential derivative with respect to the gate voltage has been calculated [72], as shown in Figure 8. Figure 8 shows that no splits and peaks exist near the flatband regions, which suggests that the analytical solution to the surface potential is better than the algorithm in the Penn State Philips (PSP) model [74].

Based on the formulas of surface potential from Chen et al., subsequently some researchers presented a complete modeling for surface potential in partially depleted poly-Si TFTs with undoped or lightly doped body by including both monoenergetic and exponential trap distributions [32]. The proposed closed-form algorithm is able to accurately calculate the surface potential and has the advantage of both accuracy and computational efficiency, which is useful for compact modeling and CAD applications.

### 3.2. Amorphous Silicon TFTs

Amorphous Silicon, especially hydrogenated amorphous-silicon (a-Si:H), has been considered as the most well-studied materials for TFTs. Generally speaking, the most important features of amorphous silicon TFT characteristics can be described by analyzing the device behavior in two regimes: below-threshold, when the electron quasi-Fermi level is in the deep states; and above-threshold, when the Fermi level enters the tail states [75]. A current model for the below- and above-threshold regimes had been proposed by considering the sheet carrier density as a function of Fermi lever position by Shur et al. [76]. In 1997, Shur et al. again developed a physically based analytical model for n-channel amorphous silicon thin film transistors and for n- and p-channel polysilicon thin film transistors, which covered all regimes of transistor operation: leakage, subthreshold, above-threshold conduction, and the kink regime in polysilicon thin film transistors [63]. Only in the last few years several models have been built, based on the description of below-threshold and above-threshold, respectively [77,78,79]. However, with gradual accumulation of the requirements imposed on the compact models and simultaneous realization of the limitations associated with the traditional modeling techniques, new physical phenomena become essential for the accurate reproduction of the device characteristics. On the other hand, due to these drawbacks in the regional approach, analytical models based on surface potential have been paid more attentions in the development of device models [40].

In terms of the consideration above, in 2008, Liu et al. presented an analytical a-Si:H TFTs model based on the surface potential [80]. In the model, when TFT is biased, the majority of the induced charges in the channel are trapped in the acceptor-like states, which divided into two groups: deep states and tail states. The distribution of localized acceptor states can be expressed as Equation (11). The localized trapped charge density is expressed as:

(19) $$n_{trapped}=\int_{-\infty }^{E_{c}}\frac{g\left(E\right)}{1+\frac{1}{g}exp\left(\frac{E-E_{f}}{k_{B}T}\right)}$$

here g is the degenerescence factor of localized states. When the density of trapped charges in the tail states are considered, the integral (Equation (19)) can be rewritten as [68] the following:

(20) $$n_{tail}=g_{t}g^{T/T_{0}}\frac{k_{B}T}{q}f\left(T,T_{t}\right)exp\frac{q\varphi -qV_{ch}\left(y\right)-E_{f0}}{k_{B}T_{0}}$$

where $g_{t}$ is the tail states density at $E_{c}$, $T_{t}$ is the tail state characteristic temperature, $V_{ch}$ stands for the channel quasi-Fermi level which is the channel voltage equal to 0 at the source and $V_{ds}$ at the drain.

To obtain the potential, the authors then solved the Poisson’s equation:

(21) $$\frac{\partial^{2}\varphi }{\varphi x^{2}}=-\frac{dF}{dx}=\frac{q}{\varepsilon_{s}}\left(n_{deep}+n_{tail}+n_{free}\right)$$

where $n_{deep}$, $n_{tail}$ and $n_{free}$ are the densities for deep trap, free, tail trap charges, respectively, $n_{free}=N_{c}exp\left(\frac{q\varphi_{s}-qV_{ch\;}\left(y\right)-E_{f0}}{k_{B}T}\right)$. According to Gauss’ law, and introducing electrical field effect, the relationship between the gate-source voltage and the surface potential can be found as follows

(22) $$C_{i}\left(V_{g}-V_{fb}-\varphi_{s}\right)=r_{t}\varepsilon_{s}exp\left(\frac{q\varphi_{s}-qV_{ch\;}\left(y\right)-E_{f0}}{k_{B}T_{0}}\right)+r_{d}\varepsilon_{s}exp\left(\frac{q\varphi_{s}-qV_{ch\;}\left(y\right)-E_{f0}}{k_{B}T_{d}}\right)+r_{f}\varepsilon_{s}exp\left(\frac{q\varphi_{s}-qV_{ch\;}\left(y\right)-E_{f0}}{k_{B}T}\right)$$

here $r_{t}=\sqrt{\frac{2k_{B}T_{0}g_{t}g^{T/T_{0}}f\left(T,T_{t}\right)k_{B}T}{q\varepsilon_{s}}}$, $r_{d}=\sqrt{\frac{2k_{B}T_{d}g_{d}g^{T/T_{d}}\pi k_{B}T}{q\varepsilon_{s}sin\left(\frac{\pi T}{T_{d}}\right)}}$ and $r_{f}=\sqrt{\frac{2k_{B}TN_{c}}{\varepsilon_{s}}}$.

To derive analytical and noniterative evaluation from Equation (22), the normalized form of Equation (22) can be written as follows:

(23) $$\left(xg-x\right)=G_{t}exp\left(x-xn\right)+G_{d}{\left[exp\left(x-xn\right)\right]}^{T_{0}/T_{d}}+G_{f}{\left[exp\left(x-xn\right)\right]}^{T_{0}/T}$$

where $xg=-\frac{V_{g}-V_{fb}}{2V_{to}}$, $x=\frac{\varphi_{s}}{2V_{to}}$, $xn=\frac{\frac{E_{f0}}{q}+V_{ch\;}\left(y\right)}{2V_{to}}$, $G_{t}=\frac{r_{t}\varepsilon_{s}}{2C_{i}V_{to}}$, $G_{d}=\frac{r_{d}\varepsilon_{s}}{2C_{i}V_{to}}$, $G_{f}=\frac{r_{f}\varepsilon_{s}}{2C_{i}V_{to}}$, and $V_{to}=\frac{k_{B}T_{o}}{q}$.

Then, by using the two-order Taylor expansion, the solution for the surface potential of amorphous silicon TFTs is expressed by:

(24) $$\varphi_{s}=x\cdot 2V_{to}.$$

**Figure 9:**
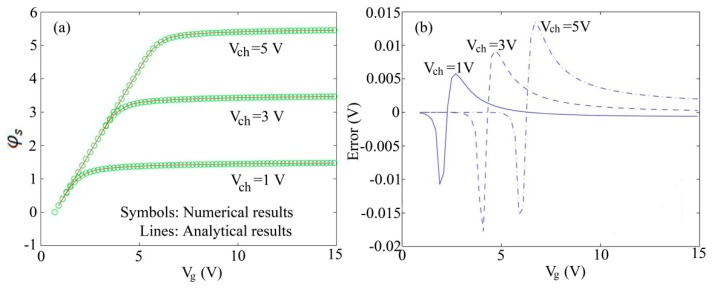
(**a**) Comparison of analytical results with the numerical results; and (**b**) absolute error of the new analytical approximation for the surface potential.

Based on the solution for the surface potential of amorphous silicon TFTs, the authors compared the analytical results with the numerical results, as shown in Figure 9a. And the absolute errors of the new analytical approximation were shown in Figure 9b. The absolute errors introduced by analytical approximation are less than 0.02 V in all cases.

After the surface potential is solved precisely, the authors then discussed the drain current by dividing the new derivation of the DC model into the below threshold region and the above threshold region.

Below threshold region, the static current of amorphous TFTs is written as:

(25) $$I_{dsd}=\mu_{n}\frac{W}{L}N_{c}\varepsilon_{s}\frac{2k_{B}T_{d}k_{B}T}{2k_{B}T_{d}-k_{B}T}{\left(\frac{1}{r_{d}\varepsilon_{s}}\right)}^{\frac{2T_{d}}{T}}C_{i}^{\frac{2T_{d}}{T}-1}\left[\frac{T}{2T_{d}}\left(\Delta \varphi_{ss}^{\frac{2T_{d}}{T}}-\Delta \varphi_{sd}^{\frac{2T_{d}}{T}}\right)+2V_{td}\frac{T}{2T_{d}-T}\left(\Delta \varphi_{ss}^{\frac{2T_{d}}{T}-1}-\Delta \varphi_{sd}^{\frac{2T_{d}}{T}-1}\right)\right]\;$$

Above threshold region, similarly, the expression of drain current in the above threshold regime can be obtained as:

(26) $$I_{dst}=\mu_{n}\frac{W}{L}N_{c}\varepsilon_{s}\frac{2k_{B}T_{0}k_{B}T}{2k_{B}T_{0}-k_{B}T}{\left(\frac{1}{r_{t}\varepsilon_{s}}\right)}^{\frac{2T_{0}}{T}}C_{i}^{\frac{2T_{0}}{T}-1}\left[\frac{T}{2T_{0}}\left(\Delta \varphi_{ss}^{\frac{2T_{0}}{T}}-\Delta \varphi_{sd}^{\frac{2T_{0}}{T}}\right)+2V_{t0}\frac{T}{2T_{0}-T}\left(\Delta \varphi_{ss}^{\frac{2T_{0}}{T}-1}-\Delta \varphi_{sd}^{\frac{2T_{0}}{T}-1}\right)\right]\;$$

**Figure 10:**
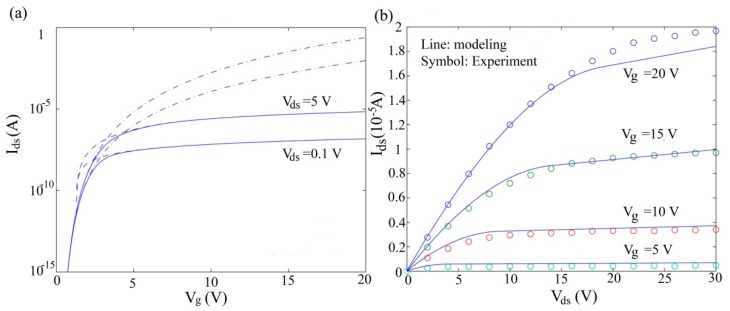
(**a**) Calculated transfer characteristics and (**b**) calculated output characteristics for a-Si:H TFT, with the measured data for comparison.

According to the expression of drain current, the calculated transfer characteristics for a-Si:H TFT is shown in Figure 10a. It is noted that a smooth transition is achieved in the below- and above-threshold regions without any use of smooth functions. Furthermore, the threshold voltage is not required in the whole calculations. Figure 10b displays the measured characteristics and the calculated current–voltage characteristics of an a-Si:H TFT. It is demonstrated that the model exhibits a reasonable agreement in both the linear region and the saturation region.

To calculate the surface potential, other methods have been used. For example, very recently, Qin et al. developed a novel scheme for surface potential of amorphous silicon TFTs by taking deep Gaussian and tail exponential distribution of the density of states into account [81]. In Qin et al.’s model, the authors adopted Taylor expansion below threshold regime, and the principle of Lamber W function and Schroder series above threshold regime, as well as Chen et al.’s model in Section 3.1.

### 3.3. OTFT Compact Models

In OTFTs, the energy disorder is usually described by Gaussian DOS as [82]:

(27) $$g\left(E\right)=\frac{N_{t}}{\sqrt{2\pi }\sigma }exp\left(-\frac{E^{2}}{2\sigma_{}^{2}}\right)$$

where *N_t_* is the total localized states, and σ indicates the width of the DOS. By connecting Gauss law $C_{i}\left(V_{g}-V_{fb}-\varphi_{s}\right)=\varepsilon_{s}F(0)$, one can obtain the following:

(28) $$C_{i}\left(V_{g}-V_{fb}-\varphi_{s}\right)=\sqrt{\frac{2q\varepsilon_{s}N_{t}}{\sqrt{2\pi }\sigma }\int_{0}^{\varphi_{s}}\int_{-\infty }^{\infty }\frac{\exp\left(-E^{2}/2\sigma^{2}\right)}{1+\exp\left(\frac{E-E_{f0}-q\left(\varphi -V\right)}{k_{B}T}\right)}dEd\varphi }$$

where *F*(0) is the electric field perpendicular to the interface at the interface, *V* is the channel voltage, and *E_f_*_0_ is the Fermi level far from the semiconductor-insulator interface.

By approximating the Fermi–Dirac distribution with the Boltzmann distribution, Equation (28) can be rewritten as:

(29) $$C_{i}\left(V_{g}-V_{fb}-\varphi_{s}\right)=\sqrt{\frac{2q\varepsilon_{s}N_{t}}{\sqrt{2\pi }\sigma }\int_{0}^{\varphi_{s}}\int_{-\infty }^{\infty }exp\left(-\frac{E^{2}}{2\sigma^{2}}-\frac{E-E_{f0}-q\left(\varphi -V\right)}{k_{B}T}\right)dEd\varphi }$$

Since the localized states mainly lie in the higher energy of Gaussian DOS, $E-E_{f0}>2k_{B}T$ is usually achieved. As the carrier density varies over a narrow range, then the Fermi–Dirac distribution can be approximated by the Boltzmann distribution. According to Equation (29), the surface potential can be calculated as:

(30) $${\left(V_{g}-V_{fb}-\varphi_{s}\right)}^{2}=kexp\left(-\frac{V}{\varphi_{t}}\right)\left(exp\left(\frac{\varphi_{s}}{\varphi_{t}}\right)-1\right)$$

where $k=\frac{\varepsilon_{0}\varepsilon_{s}k_{B}N_{t}}{C_{i}{}^{2}}exp\left(-0.5\sigma^{2}\right)$. The solution of Equation (30) actually is numerical. However, under low gate voltage OTFTs operate in weak accumulation mode, that is, $\varphi_{s}\ll \varphi_{t}$. In this situation, the surface potential $\varphi_{sw}$ is small and can be obtained as

(31) $$\phi_{sw}=V_{g}-V_{fb}+\frac{kexp\left(-\frac{V}{\varphi_{t}}\right)}{2\varphi_{t}}-\sqrt{{\left(V_{g}-V_{fb}+\frac{kexp\left(-v/\varphi_{t}\right)}{2\varphi_{t}}\right)}^{2}-\left(V_{g}-V_{fb}\right)}$$

Under high gate voltage, OTFTs operate in a strong accumulation mode, that is, $V_{g}-V_{fb}\gg \varphi_{s}\gg \varphi_{t}$. In this case, the surface potential $\varphi_{ss}$ reads as

(32) $$\phi_{ss}=2\varphi_{t}ln\left(\frac{V_{g}-V_{fb}}{\sqrt{k}}\right)+V$$

Connecting Equations (31) and (32), the unified surface potential of OTFTs is expressed as

(33) $$\varphi_{s}=\sqrt{\frac{\varphi_{sw}{}^{\gamma }\cdot \varphi_{ss}{}^{\gamma }}{\varphi_{sw}{}^{\gamma }+\varphi_{ss}{}^{\gamma }}}$$

**Figure 11:**
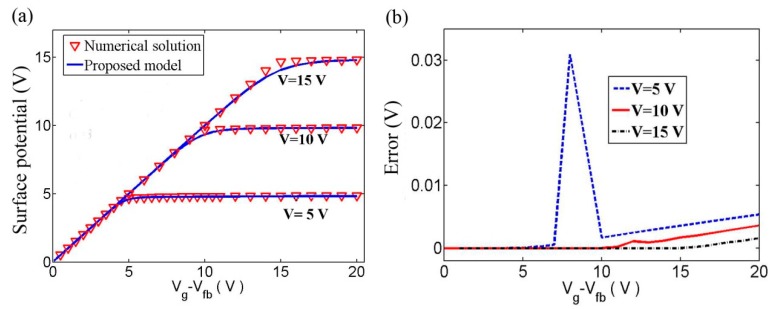
(**a**) Comparison between the surface potential calculated using Boltzmann distribution and Fermi–Dirac distribution functions for different channel voltages and (**b**) absolute error of the Boltzmann approximation from (**a**).

Figure 11a shows the comparison between the surface potential calculated using the Boltzmann distribution and Fermi–Dirac distribution functions under different channel voltages, respectively [83]. One can see that a good agreement is observed. Figure 11b shows the absolute and relative error of the Boltzmann function approximation from Figure 11a, revealing that the maximum of relative error is less than 0.6%, as shown by the maximum peak in Figure 11b. This approximation displays good accuracy for weak, moderate and strong accumulation at various channel voltages. Otherwise, the absolute error of the surface potential introduced by the Boltzmann function approximation decreases with channel voltage and is always lower than 0.035 V.

For OTFTs, the field-effect mobility can be written as [84]:

(34) $$\mu =\mu_{0}exp\left(C_{1}{\left(2n/N_{t}\right)}^{C_{2}}\right)$$

here *C*_1_ and *C*_2_ are given as $C_{1}=0.5\left(S^{2}-S\right)$ and $C_{2}=2\frac{ln\left(S^{2}-S\right)-ln\left(ln\left(4\right)\right)}{S^{2}}$, which only depend on the disorder, *n* is the carrier concentration, $\mu_{0}=\mu_{00}exp\left(-aS-bS^{2}\right)$, *S* = *σ*/*k_B_T*, and $\mu_{00}$ is the mobility in the limit *n* → 0.

Using the same method in the literature [85], the field-effect mobility $\mu_{eff}$ is calculated with the following:

(35) $$\mu_{eff}=\frac{L}{C_{i}WV_{ds}}\frac{\partial I_{ds}}{\partial V_{g}}=\frac{\sqrt{\varepsilon_{s}\varepsilon_{0}/2q}}{C_{i}}\frac{n(\varphi_{s})\mu (n(\varphi_{s}))}{\sqrt{\int_{0}^{\varphi }\int_{-\infty }^{\infty }g(E)dEd\varphi }}\times \frac{2C_{i}\sqrt{\int_{0}^{\varphi_{s}}\int_{-\infty }^{\infty }g(E)dEd\varphi }}{\sqrt{\varepsilon_{s}q}n(\varphi_{s})}\approx \mu_{0}exp(C_{1}\frac{{\left(2C_{i}\right)}^{2C_{2}}}{{\left(2\varepsilon_{s}k_{B}TN_{t}\right)}^{C_{2}}}{\left(V_{g}-V_{fb}-\gamma V_{ds}\right)}^{2C_{2}})$$

where $n(\varphi_{s})=\int_{-\infty }^{\infty }g(E){\left(1+exp\left(\frac{E-E_{f0}-q(\varphi -V)}{k_{B}T}\right)\right)}^{-1}dE$, γ is a parameter that accounts for channel-length modulation.

Then, according to Gauss’s law, the sheet density of total induced charges in the channel is given by:

(36) $$Q_{i}=C_{i}\left(V_{g}-V_{fb}-\phi_{s}\right)\approx C_{i}\sqrt{K}exp\left(\frac{\left(\varphi_{s}-V\right)}{2\varphi_{t}}-1\right)$$

By differentiating Equation (36) with respect to *φ_s_*, we then obtain:

(37) $$\frac{dV}{d\varphi_{s}}=\frac{2\varphi_{t}}{\sqrt{K}}exp\left(-\frac{\varphi_{s}-V}{2\varphi_{t}}+1\right)+1=2\varphi_{t}\frac{C_{i}}{Q_{i}}+1$$

Using the gradual channel approximation, $I_{ds}$ is given by:

(38) $$I_{ds}=-\mu_{eff}WQ_{i}\frac{dV}{dy}=-\mu_{eff}WQ_{i}\left(2\varphi_{t}\frac{C_{i}}{Q_{i}}+1\right)\frac{d\varphi }{dy}$$

By integrating Equation (38) from $\varphi_{s}=\varphi_{ss}$ to $\varphi_{s}=\varphi_{sd}$, the static current of OTFTs becomes:

(39) $$I_{ds0}=\frac{\mu_{eff}W}{L}\left(2\varphi_{t}C_{i}\left(\varphi_{sd}-\varphi_{ss}\right)-\frac{C_{i}}{2}\left({\left(V_{g}-V_{fb}-\varphi_{sd}\right)}^{2}-{\left(V_{g}-V_{fb}-\varphi_{ss}\right)}^{2}\right)\right)$$

where $\varphi_{ss}$ and $\varphi_{sd}$ are the surface potentials at the source and drain side, respectively. Both of them can be analytically calculated by Equation (33). When OTFTs are biased to the saturation region, channel-length modulation becomes significant in short channel devices. In this case, the expression of *I_ds_* can be rewritten as:

(40) $$I_{ds}=I_{ds0}\left(1+\lambda V_{ds}\right)$$

Based on Equation (40), the OTFT characteristics can be described by a new formula that does not contain the threshold voltage.

**Figure 12:**
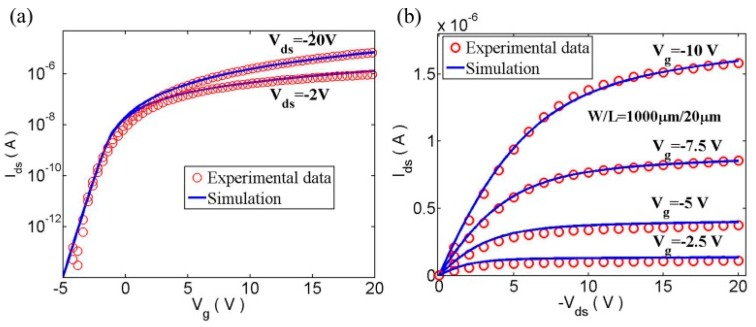
(**a**) Simulated and experimental results for transfer characteristics of organic Thin-film transistors (OTFT); and (**b**) comparison between the simulated and experimental results for output characteristics of OTFT for different gate voltages.

Figure 12 shows the measured characteristics from pentacene transistors and the calculated current–voltage characteristics of OTFT. The model agrees well with the experimental results in both the linear and saturation regions [83].

**Figure 13:**
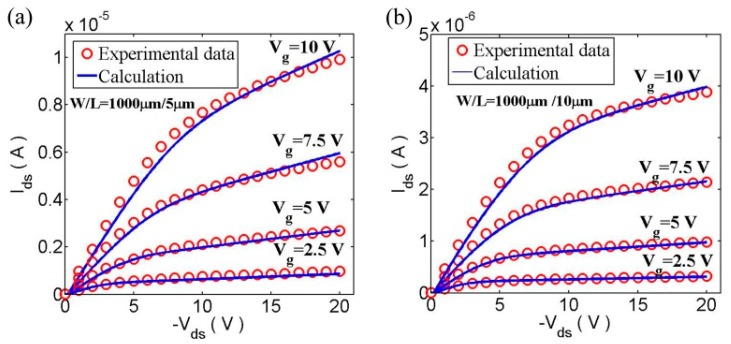
Simulated and experimental results of output characteristics of OTFT: (**a**) for W/L = 1000 μm/5 μm and (**b**) for W/L = 1000 μm/10 μm.

We also verified our proposed model by comparing it to measurements of OTFTs with channel lengths from 25 µm to 5 µm (*W* = 1000 µm), as shown in Figure 13 [83]. The extracted λ values are 0.55 and 0.27 for *L* = 5 μm and *L* = 10 μm, respectively.

### 3.4. a-IGZO TFTs

As mentioned in Section 2.3, the MTR theory is responsible for the charge transport of a-IGZO TFTs. We have combined the MTR theory with the surface potential to develop the compact model of IGZO TFTs [86,87]. Generally speaking, in TFTs, due to the accumulated carriers in semiconductor-insulator interface under the gate voltage, the gate-induced potential *φ*(*x*) shifts the difference between the mobility edge and the Fermi level. The quasi-Fermi level *E_f_*(*x*) is

(41) $$E_{f}\left(x\right)=E_{f0}+q\varphi \left(x\right)$$

The variation of *φ*(*x*) with respect to the distance *x* is determined by the Poisson equation as [57]:

(42) $$F{\left(x\right)}^{2}=\frac{2q}{\varepsilon_{s}}n_{total}=\frac{2q}{\varepsilon_{s}}\left[N_{t}v_{0}\tau_{0}exp\left(\frac{E_{f}\left(x\right)}{k_{B}T}\right)+\overset{\varphi \left(x\right)}{\underset{0}{\int }}\overset{0}{\underset{-\infty }{\int }}\frac{g\left(E\right)}{1+exp\left(\frac{E-E_{f}\left(x\right)}{k_{B}T}\right)}dEd\varphi \left(x\right)\right]$$

where *F*(*x*) is the electric field perpendicular to the interface. At the interface, the electric field *F*(0) can be expressed through Gauss’s law as:

(43) $$\varepsilon_{s}F\left(0\right)=C_{i}\left(V_{g}-V_{fb}-\varphi_{s}\right)=\sqrt{2q\varepsilon_{s}\left[N_{t}v_{0}\tau_{0}exp\left(\frac{E_{f}\left(x\right)}{k_{B}T}\right)+\int_{0}^{\varphi \left(x\right)}\int_{-\infty }^{0}\frac{g\left(E\right)}{1+exp\left(\frac{E-E_{f}\left(x\right)}{k_{B}T}\right)}dEd\varphi \left(x\right)\right]}$$

where *T_TA_* is the characteristic temperature of the exponential DOS, $\tau_{0}$ is the lifetime of carriers, and $v_{0}$ is the attempt-to-escape frequency. Then, the field effect mobility could be written as [88]:

(44) $$\mu_{eff}=\frac{\mu_{e}}{1+{\left(\frac{1}{v_{0}\tau_{0}}\Gamma \left(1+T/T_{TA}\right)\Gamma \left(1-T/T_{TA}\right)exp\left(\frac{E_{f0}+q\varphi_{s}}{k_{B}T_{TA}}\left(\frac{T}{T_{TA}}-1\right)\right)\right)}^{-1}}$$

where $\mu_{e}$ is the band mobility and Γ(z)=πz/sinπz. Under the low gate voltage, Fermi level lies in the deep states and hence free carriers above the mobility edge can be neglected, and carriers of localized states will dominate the transport of IGZO TFTs (corresponding to the sub-threshold regime of transistor). Thus, the total carrier concentration is reasonably written as

(45) $$n\left(x\right)\approx \int_{-\infty }^{0}\frac{g\left(E,x\right)}{1+exp\left(\frac{E-E_{f}\left(x\right)}{k_{B}T}\right)}dE=N_{t}\Gamma \left(1+\frac{T}{T_{TA}}\right)\Gamma \left(1-\frac{T}{T_{TA}}\right)exp\left(\frac{E_{f0}+q\varphi_{s}}{k_{B}T_{TA}}\right)$$

Substituting Equations (45) into (43), one can get the following expression:

(46) $$C_{i}\left(V_{g}-V_{fb}-\varphi_{s}\right)=\sqrt{q\varepsilon_{s}\left[N_{t}v_{0}\tau_{0}exp\left(\frac{E_{f}\left(x\right)}{k_{B}T}\right)+N_{t}\Gamma \left(1+\frac{T}{T_{TA}}\right)\Gamma \left(1-\frac{T}{T_{TA}}\right)exp\left(\frac{E_{f0}+q\varphi_{s}}{k_{B}T_{TA}}\right)\right]}$$

To achieve the analytic solution of the surface potential, we transformed Equation (46) as:

(47) $$V_{g}-V_{fb}-\varphi_{s}=G_{T}exp\left(\frac{q\varphi_{s}-qV_{ch}}{2k_{B}T}\right)+G_{TA}exp\left(\frac{q\varphi_{s}-qV_{ch}}{2k_{B}T_{TA}}\right)$$

$G_{T}$ and $G_{TA}$ can be expressed as:

(48) $$\{\begin{array}{l} G_{T}=\frac{1}{C_{i}}\sqrt{q\varepsilon_{s}v_{0}\tau_{0}N_{t}exp\left(\frac{E_{f0}}{k_{B}T}\right)}\\ G_{TA}=\frac{1}{C_{i}}\sqrt{q\varepsilon_{s}N_{t}\Gamma \left(1+\frac{T}{T_{TA}}\right)\Gamma \left(1-\frac{T}{T_{TA}}\right)exp\left(\frac{E_{f0}}{k_{B}T_{TA}}\right)} \end{array}$$

Through estimating the order of magnitudes, in Equation (47) the first term is much smaller than the second term. Thus, we only consider the second term and ignore the first term. By using two-order Taylor expansion, one can get:

(49) $$x_{i}=xg\left\{{\left[{\left(xg+1\right)}^{2}+2xn+2log\left(\frac{xg}{G_{T}}\right)\right]}^{1/2}-xg-1\right\}$$

However, if one considers only the second term in Equation (47), some errors in the surface potential calculation maybe occur. In order to improve the accuracy, we add some corrections by using the Schroder series method to cover the influence of the first term in Equation (47). Finally, the analytical solution of the surface potential can be written as:

(50) $$\{\begin{array}{l} \varphi_{s}=2\frac{k_{B}T_{TA}}{q}\left[x_{i}-\frac{f}{\partial f}\left(1+\frac{\partial^{2}f}{2\partial f}\frac{f}{\partial f}\right)\right]\\ f=\left(xg-x\right)-G_{TA}exp\left(x-xn\right)-G_{T}{\left(exp\left(x-xn\right)\right)}^{\frac{T_{TA}}{T}} \end{array}$$

**Figure 14:**
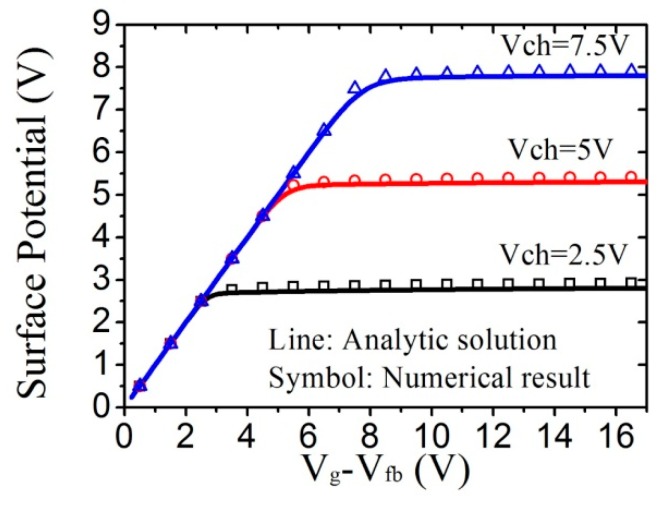
Comparison of the calculated surface potential between analytic solution and the numerical results for different channel voltages.

Figure 14 shows a comparison of calculated surface potential between analytic solution and numerical result [86,87]. The percentage error between the numerical and analytical solutions is always below 0.2%. The parameters are *T* = 300 K, *T_TA_* = 405 K, *ν*_0_*τ*_0_ = 1, *V_fb_* = 0.5 V, *C_i_* = 8.85 × 10^−8^ F/cm^2^, and *μ_e_* = 19.7 cm^2^/Vs.

Using the gradual channel approximation, the current equation is given as:

(51) $$I_{ds}=-\mu_{eff}WQ_{i}\frac{dV}{dy}=-\mu_{eff}WQ_{i}\left(2\varphi_{t}\frac{C_{i}}{Q_{i}}+1\right)\frac{d\varphi }{dy}$$

By integrating Equation (51) from *φ_s_* = *φ_ss_* to $\varphi_{s}=\varphi_{sd}$, the static current of a-IGZO TFTs is expressed as:

(52) $$I_{ds0}=\mu_{eff}\frac{W}{L}\left[2\varphi_{t}C_{i}\left(\varphi_{sd}-\varphi_{ss}\right)-\frac{1}{2}\left({\left(V_{g}-V_{fb}-\varphi_{sd}\right)}^{2}-{\left(V_{g}-V_{fb}-\varphi_{ss}\right)}^{2}\right)\right]$$

where $\varphi_{ss}$ and $\varphi_{sd}$ are the surface potential at source and drain side, respectively. Both of them can be analytically calculated from Equation (50).

**Figure 15:**
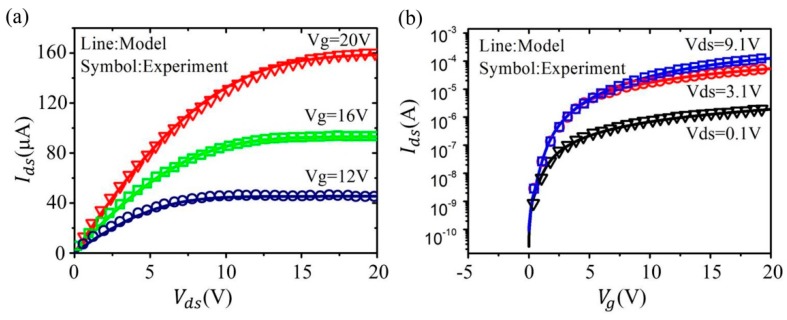
Comparison between the calculation and experimental data; (**a**) for output characteristics of In-Ga-Zn-O (IGZO) under different gate voltages; and (**b**) transfer characteristics of IGZO under different drain-source voltages.

**Figure 16:**
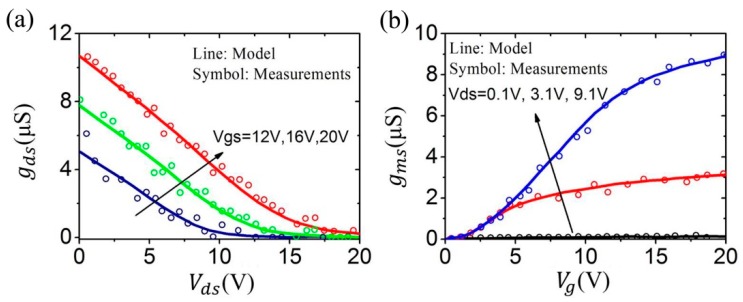
Model $g_{ds}-V_{ds}$ curves (**a**) and trans-conductance curves (**b**).

Figure 15 shows the output and transfer characteristics curve. The good agreement between our modeling results and the experimental data has been observed [86,87]. Figure 16 shows the drain conductance and trans-conductance curves [86,87]. Our model well agrees with the measured results.

## 4. Comparison of Various Compact Models

As mentioned above, the most difference between silicon-based TFTs and TFTs with new active material (e.g., OTFTs and IGZO TFTs) derived from the fact that whether the threshold voltage can be defined in TFT device. Since OTFTs and IGZO TFTs usually operate in accumulation region, the formulation of compact model should discard the influence of the threshold voltage. The following will give a comparison for different compact models.

### 4.1. Comparison of Model Accuracy

**Figure 17:**
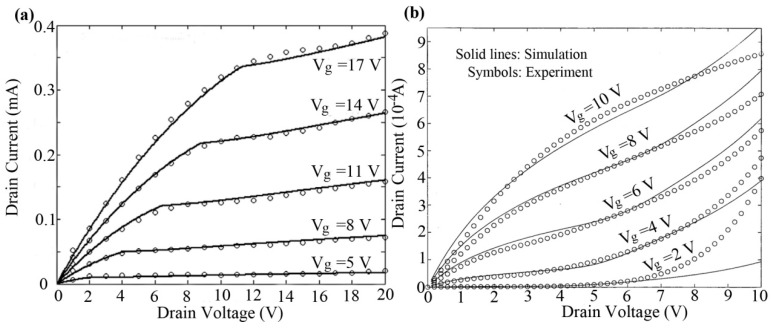
Comparison of $V_{ds}-I_{ds}$ of polysilicon TFTs based on the surface-potential from Chen et al. (**a**), and the effective medium approximation from Iñiguez et al. (**b**).

**Figure 18:**
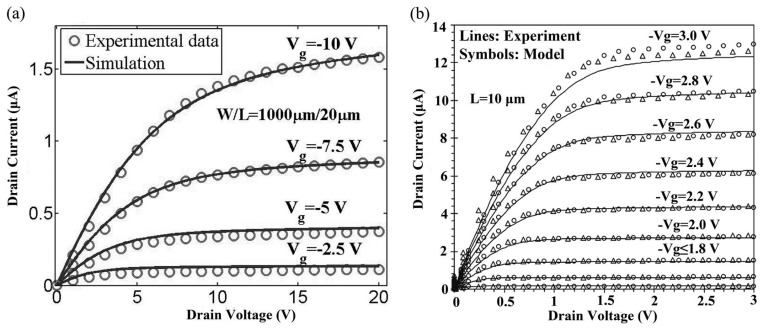
Comparison of $V_{ds}-I_{ds}$ of OTFTs based on the surface-potential (**a**), and the generic model (**b**), respectively.

For the compact models, the central aim is to accurately and physically describe the current–voltage characteristics of TFTs. Here, we will discuss various compact models and their accuracies verified for TFTs. For polysilicon TFTs, we firstly compare $V_{ds}-I_{ds}$ characteristics based on the surface-potential-based by Chen et al. [72] and the EMA method by Iñiguez et al. [28], respectively, as shown in Figure 17. It is obvious that the surface-potential-based model agrees well with experimental data. However, the simulated results from Iñiguez et al. show a well consistent between model and experiment under low drain voltage, with increasing the drain voltage, the model seriously deviated from the experiment. Similar errors of model accuracy have also been found in the OTFT compact models. Figure 18 shows a comparison of $V_{ds}-I_{ds}$ characteristics based on the surface potential and the generic model, respectively [24,83]. For the surface-potential-based model in Figure 18a, the accuracy is good in all regions, but for the generic model in Figure 18b the errors increase with the gate voltage increasing.

Strictly speaking, the errors derive from the transformation from the numerical equation to the analytical solution. To obtain analytical solution, the authors usually transferred the numerical model to analytical expression by using some reasonable assumption. Intuitively, various assumptions will generate different error values, which finally affect the model accuracy. Thus, the analysis of the error is essential to transfer the numerical equation to the analytical expression. For the surface-potential-based compact models, reducing the errors of the calculated surface potential has become an important criterion. However, as compared with surface-potential-based models, the errors for the charge-based models are always ignored, which thus results in the lower accuracy.

### 4.2. Parameter Comparison and Extraction

Apart from the accuracy and comprehensive nature, an excellent compact model should include as few parameters as possible fitting the TFT characteristics. Table 1 and Table 2 give a summary of the parameter comparison for the surface-potential-based compact models and OTFT compact models based on different approaches, respectively. It is found that the researchers always aspired for as few parameter numbers as possible during developing compact models. Actually, for the compact model, the fewer the non-physical parameters (fitting parameters), the better the model is considered. From Table 1 and Table 2, one can see that the parameter numbers in our model is just 12, which is superior, compared with other models.

**Figure 19:**
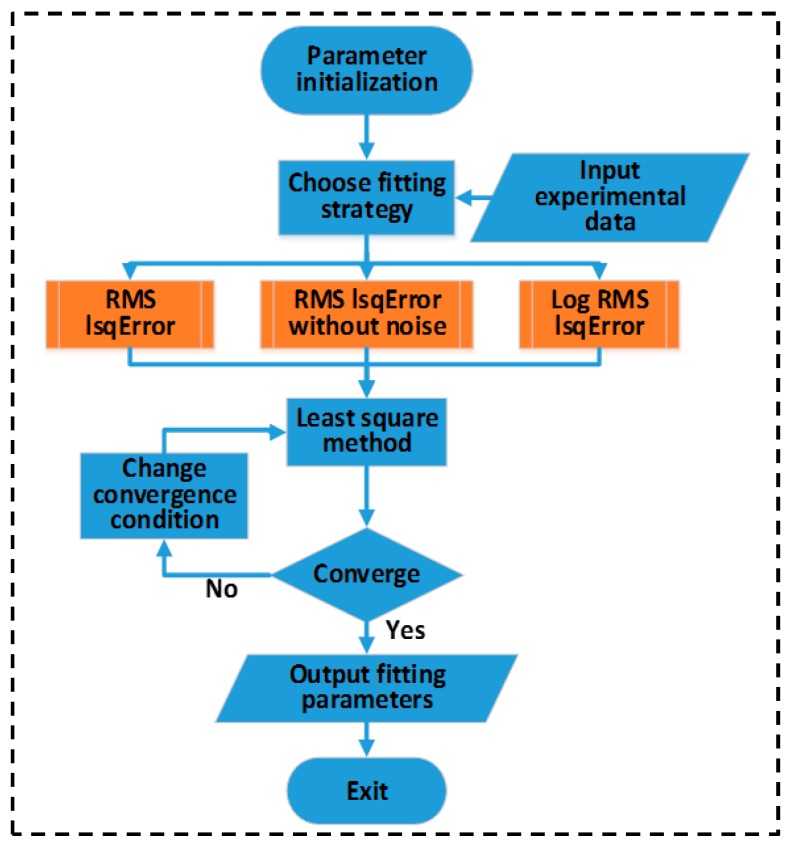
Extraction flow of key physical parameters of the model.

In addition to using as few parameters as possible, parameter extraction also plays an important role in understanding TFT characteristics. Generally speaking, parameter extraction aims at being physical. To achieve higher level, the parameter sequence should introduce physical effect [21]. However, considering the continuity and accuracy of compact model, the fitting parameters will be used for smoothing the output curves and reducing the error. It is anticipated that the compact models of TFTs with the parameter setting will be suitable to circuit design and can provide accurate insight into the performance. The main criterion for a good set of parameters is the balance of error, efficiency and continuity. For IGZO TFTs, we have developed an extraction flow of the key physical parameters of the surface-potential-based compact model, as shown in Figure 19 [86,87]. Based on the corresponding equations shown in Figure 19, four key parameters can be extracted, that is, the maximum mobility $\mu_{0}$, the characteristic temperature $T_{TA}$, the product of the escape frequency $v_{0}$ and carrier lifetime $\tau_{0}$.

### 4.3. Criterion and Continuous Test of Compact Models

A compact model must satisfy several rather restrictive requirements imposed by their use in advanced circuit simulators. From the mathematical point of view, the equations of the models should meet three classes at least [40], that is, “class 1” in order to be compatible with Newton–Raphson-based circuit simulators, with “class 2” or better preferred in order to achieve faster convergence, and “class 3” required for circuit simulation of active-matrix organic light-emitting diode (AMOLED) displays or distortion modeling in RF circuits. Currently, the most compact models are satisfied to the “class 1”. A small numbers of compact models can meet the requirements of “class 2” and “class 3” together. For the “class 3” requirement, the application is completely based on the active layers of TFTs. For example, silicon-based TFTs (poly-Si and a-Si:H) are mainly used in AMOLED displays. OTFTs can be applied to logic circuit design and flat-panel display. IGZO TFTs can be used in constructing RFID tags or inverter. Thus, the compact model of TFTs should be established according to their application.

**Figure 20:**
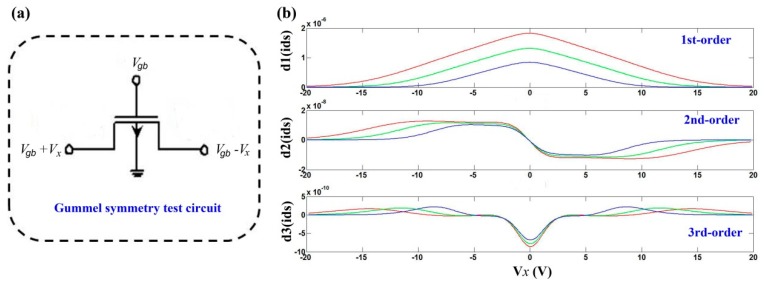
(**a**) Gummel symmetry test circuit for IGZO TFTs; and (**b**) Gummel symmetry test for the 1, 2, 3-order derivative of the drain current under different gate voltages.

In addition, it would be specially mentioned that, in order to meet the requirement of “class 2”, the compact model must fulfill one of the benchmark tests, i.e., Gummel symmetry test (GST) [21,91,92]. Based on our surface-potential-based compact model for IGZO TFTs, the GST has been provided [86,87], as shown in Figure 20. Figure 20a shows a GST circuit for IGZO TFTs. Generally, the higher-order derivatives in TFT compact models are obtained as a function of $V_{x}$, which is symmetry for $V_{x}=0$. This symmetry roots in the symmetry device structure and channel. Figure 20b shows the GST for the 1, 2, 3-order derivative of the drain current of IGZO TFTs, which display a good continuity and symmetry. Thus, our compact model in IGZO TFTs can pass the GST.

## 5. Conclusions and Outlook

Compact models form a critical link between the manufacturing teams and the chip design teams by mathematically capturing the properties of devices. We have reviewed the concept, development and application of compact model of TFTs. Based on different active materials in TFTs, the charge transport characteristics has also been discussed in detail. Based on the different approaches, especially the surface-potential-based, the merits and shortcomings for current compact models have discussed. We also proposed our surface-potential-based compact models for organic and IGZO TFTs and parameter extraction technology. The comparison of various compact models has been summarized.

Currently, the compact model is still open and evolving. To achieve the excellent compact model, the following should be considered: accurate in all regions of operation and types, suitable for all simulation modes, excellent convergence, and intuitive and easy to extract parameters. In addition, to keep pace with the increase of circuit operating frequencies and device tolerances scale down, the compact model of TFTs should account for the bias dependent contact resistances, gate tunneling, interface effect and scaling effect. The dynamic behavior, aging and hysteresis of TFTs also should be considered in developing the compact model to pursue the future circuit design.

## Figures and Tables

**Table 1:** Comparison of fitting parameter numbers for the surface-potential-based compact models.

Types	Parameter Numbers	Authors	Years
Polysilicon TFTs	15 [67]	Y. Shimizu, et al.	2006
	14 [72]	R. S. Chen, et al.	2007
	11 [32]	W. L. Deng, et al.	2011
Amorphous TFTs	16 [80]	Y. Liu, et al.	2008
	22 [89]	Y. Liu, et al.	2009
	14 [81]	J. Qin, et al.	2014
Organic TFTs	12 [83]	Our work	2015
IGZO TFTs	14 [90]	A. Tsormpatzoglou, et al.	2013
	12 [86]	Our work	2014

**Table 2:** Comparison of fitting parameter numbers for OTFT compact model based different approaches.

Years	Parameter Numbers	Authors	Method
1995	14 [39]	M. S. Shur, et al.	Effective medium approach
1999	28 [27]	B. Iñiguez, et al.	Effective medium approximation
1999	14 [29]	M. D. Jacunski, et al.	Semi-empirical approach
2006	15 [67]	Y. Shimizu, et al.	Surface potential
2007	14 [72]	R. S. Chen, et al.	Surface potential
2007	12 [30]	W. J. Wu, et al.	Generation-recombination model
2011	11 [32]	W. L. Deng, et al.	Surface potential
2015	12 [83]	Our work	Surface potential
